# Correction: Voltage-dependent inward currents in smooth muscle cells of skeletal muscle arterioles

**DOI:** 10.1371/journal.pone.0203342

**Published:** 2018-08-28

**Authors:** The PLOS ONE Staff

The following information is missing from the Funding section: The National Institute of Health, R01 MH079406 (RES) and New Jersey Health Foundation (RES). The funders had no role in study design, data collection and analysis, decision to publish, or preparation of the manuscript.

The publisher apologizes for the error.
