# Supplementary figures and images for: Voltage-dependent inward currents in smooth muscle cells of skeletal muscle arterioles
Source: PLoS One. 2018 Apr 25;13(4):e0194980. doi: 10.1371/journal.pone.0194980 (PMC5919073; doi:10.1371/journal.pone.0194980)

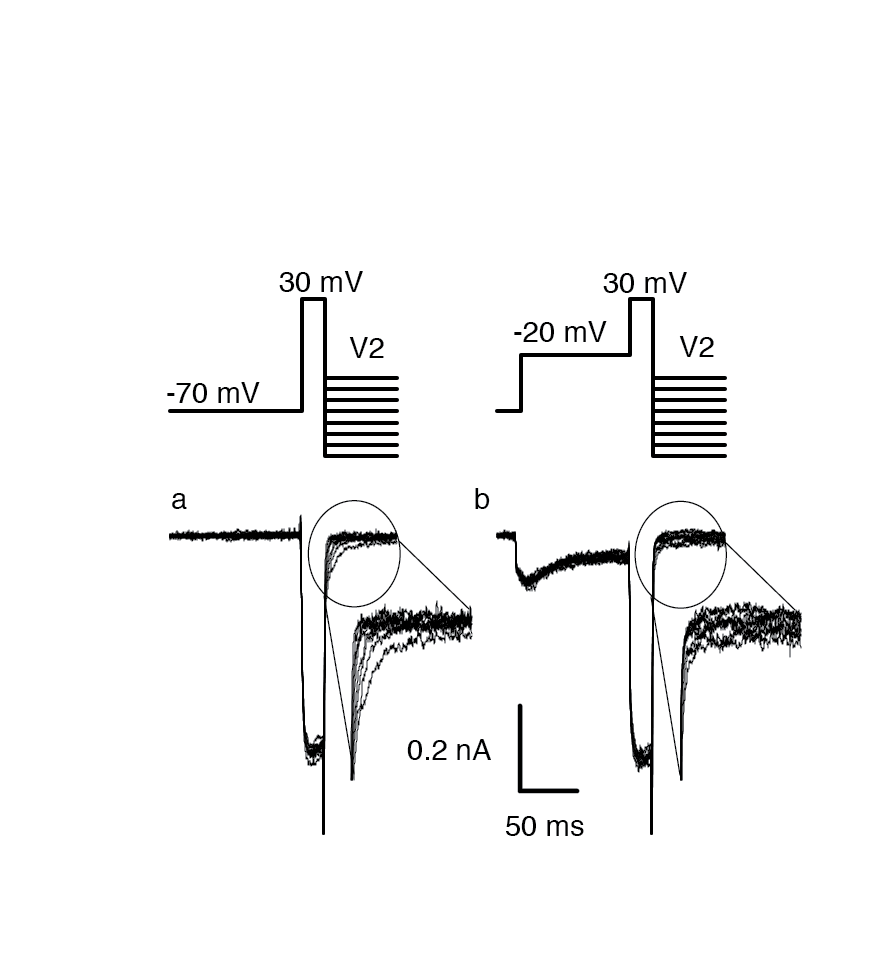

Supplement: S1 Fig — The tail currents elicited after activating step to 30 mV had significant slow component (tracings a). The slow component was absent if the conditioning that inactivates currents at –20 mV preceded the activating step (tracings b). Similar observations were done on three cells (n = 3). (TIF) [file pone.0194980.s001.tif]

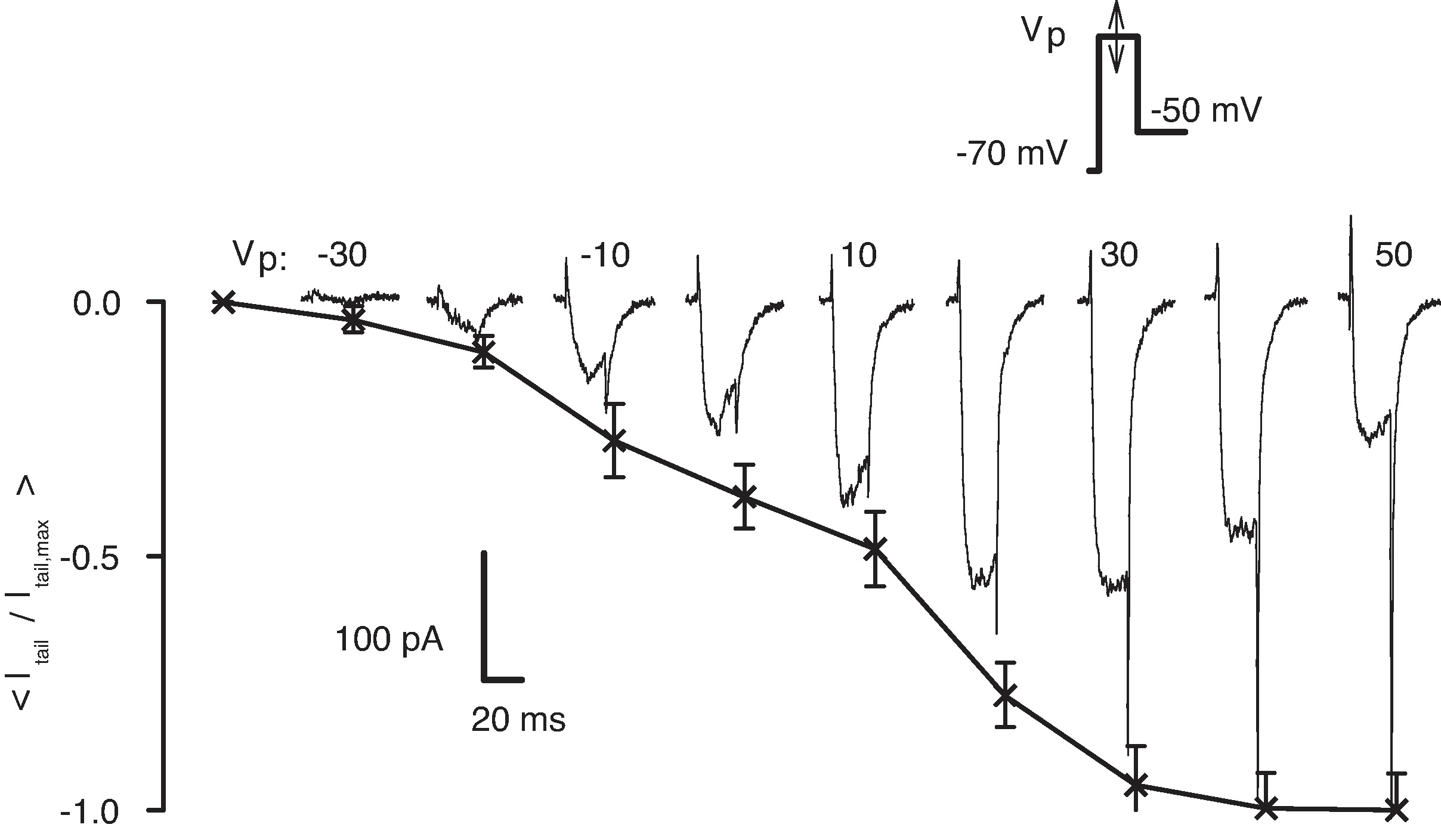

Supplement: S2 Fig — Test voltage protocol is shown at the top. Traces of representative currents for different pre-pulse are shifted along the x-axis by intervals proportional to the pre-pulse voltage (indicated). Before averaging (n = 3), amplitudes of the tail current in each cell were normalized to their value for activation step to 50 mV. (TIF) [file pone.0194980.s002.tif]

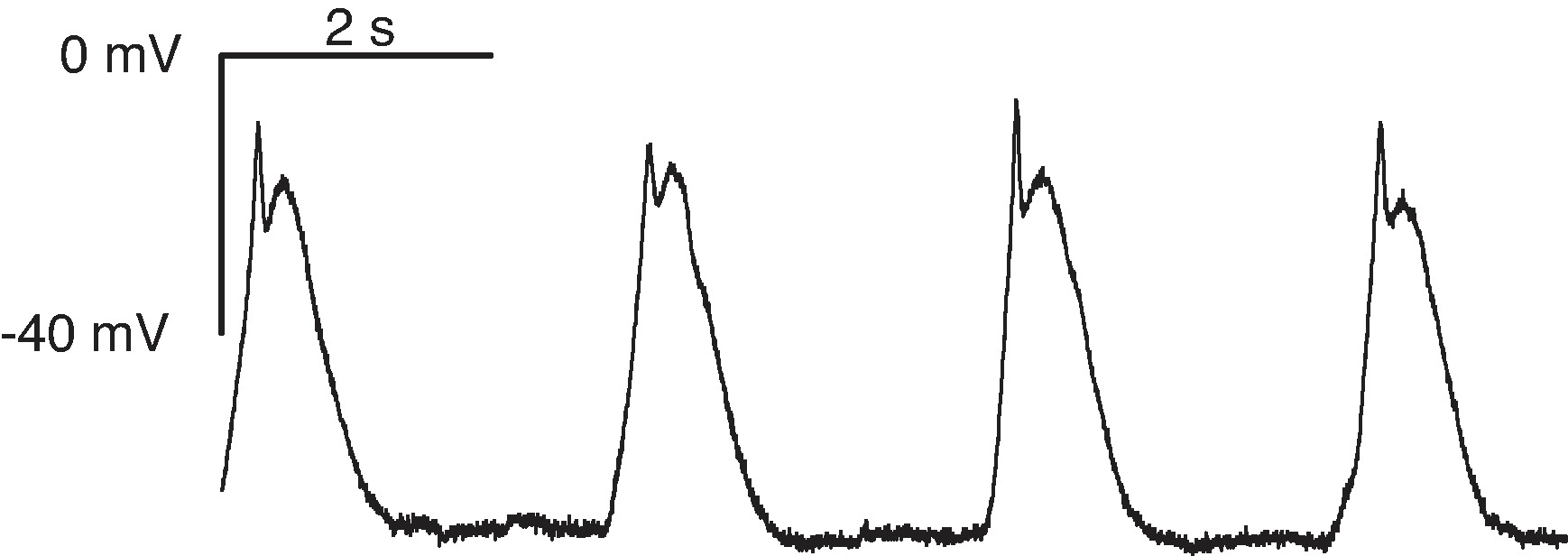

Supplement: S3 Fig — Recording conditions: gramicidin-perforated patch-clamp with 2Ca bathing solution and 150 mM KCl in the pipette. Similar rhythmic activity was observed in six out of 81 cells studied. Most cells had stable membrane potential (n = 6). (TIF) [file pone.0194980.s003.tif]

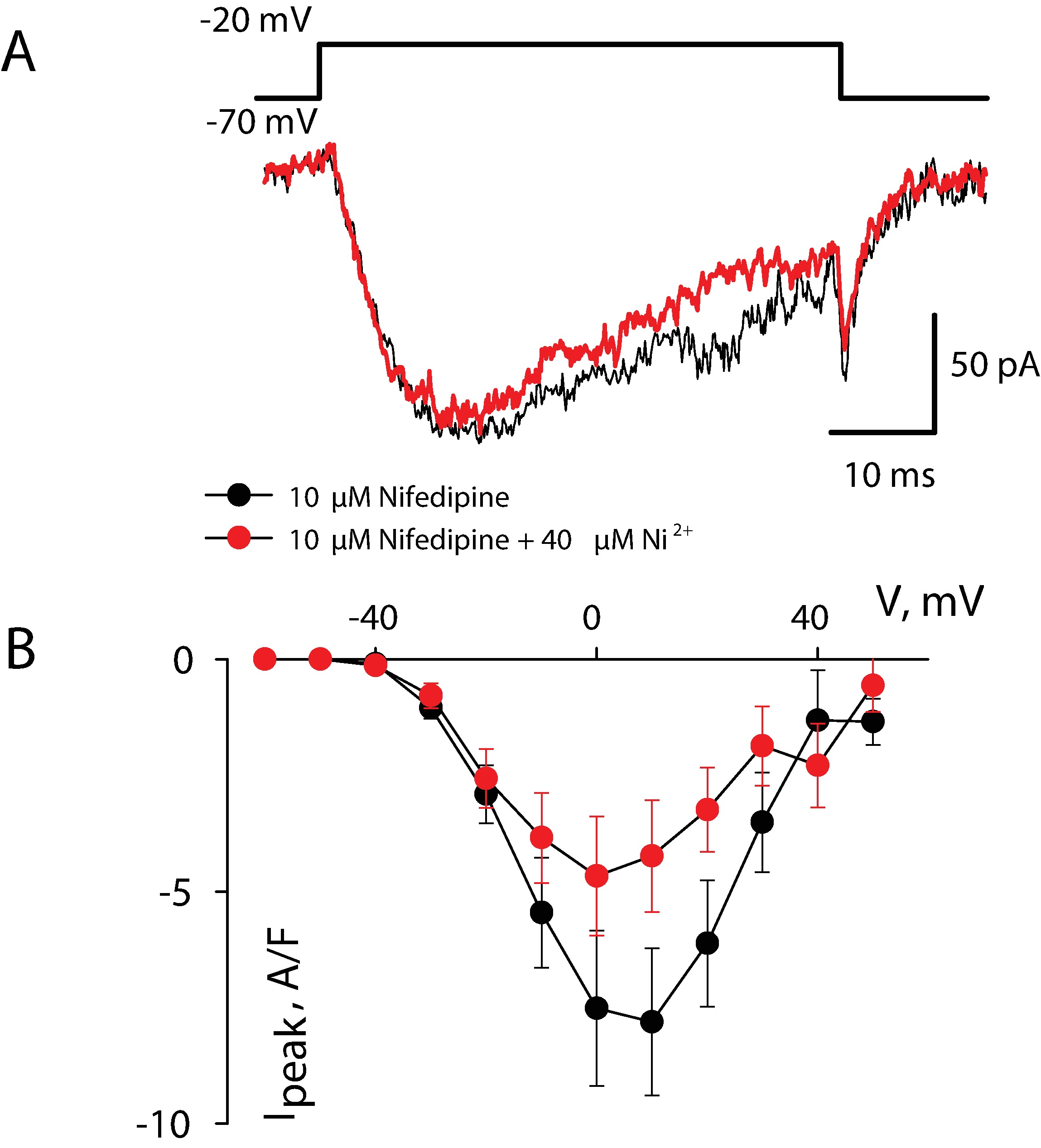

Supplement: S4 Fig — A, T-type Ca2+ currents were recorded in 2Ca solution in the presence of 1 μm TTX and 10 μm Nifedipine, with and without 40 μm Ni2+. B, Averaged peak current-voltage relationships (n = 5). Application of 40 μm Ni2+ did not have a significant effect on the magnitude of T-type Ca2+ currents. (TIF) [file pone.0194980.s004.tif]
